# Persistence of respiratory and inflammatory responses after dermal sensitization to persulfate salts in a mouse model of non-atopic asthma

**DOI:** 10.1186/s13223-016-0131-3

**Published:** 2016-05-24

**Authors:** M. J. Cruz, M. Olle-Monge, J. A. Vanoirbeek, A. Assialioui, S. Gomez-Olles, X. Muñoz

**Affiliations:** Servicio de Neumologia, Hospital Universitario Vall d’Hebron, Passeig Vall d’Hebron, 119, 08035 Barcelona, Spain; CIBER Enfermedades Respiratorias (CibeRes), Barcelona, Spain; Departament de Medicina, Universitat Autònoma de Barcelona, Barcelona, Spain; Centre of Environment and Health, KU Leuven, Leuven, Belgium; Department of Cell Biology, Physiology and Immunology, Universitat Autònoma de Barcelona, Barcelona, Spain

**Keywords:** Occupational asthma, Airway hyperresponsiveness, Lung inflammation, Ammonium persulfate

## Abstract

**Background:**

Exposure to ammonium persulfate (AP) has been reported to be the main cause of occupational asthma in hairdressers. The aim of this study is to assess how long the asthmatic response to AP can be induced after dermal sensitization in a mouse model.

**Methods:**

BALB/c mice received dermal applications of AP or dimethylsulfoxide (DMSO) (control) on days 1 and 8. They then received a single nasal instillation (challenge) of AP or saline on days 15, 22, 29, 36, 45, 60 and 90. Respiratory responsiveness to methacholine was measured 24 h after the challenge using a non-specific methacholine provocation test. Pulmonary inflammation was analysed in bronchoalveolar lavage (BAL), and total serum immunoglobulin (Ig) E, IgG1 and IgG2a were measured in serum samples. Histological analysis of lung slides was performed.

**Results:**

Mice dermally sensitized and intranasally challenged with AP showed respiratory responsiveness to methacholine as long as 45 days after initial sensitization, as well as increased percentage of neutrophils in BAL compared with the control group. At day 60, dermally sensitized mice still presented bronchial hyperresponsiveness, while the percentage of neutrophils returned to baseline levels similar to those of controls. Total serum IgE increased significantly on day 22 after dermal sensitization. Total serum IgG1 and IgG2a increased from 45 days after dermal sensitization and remained high at 90 days.

**Conclusions:**

Both respiratory responsiveness to methacholine and airway inflammation responses decrease with increasing time between sensitization and challenge. Respiratory responsiveness to methacholine tends to persist longer than inflammation.

## Background

Persulfate salts are highly reactive low molecular weight (LMW) chemical compounds which are present in considerable proportions (10–20 %) in the bleaching powders used by hairdressers during hair-bleaching procedures [1]. Exposure to these salts has been identified as the main cause of immunological sensitization and subsequent allergic diseases such as contact dermatitis and bronchial asthma, and it has been associated with a high risk of occupational asthma (OA) in hairdressers [2–4].

Nevertheless, the mechanisms by which persulfate salts induce sensitization and OA are not well established [5]. An immunologic mechanism has been postulated; various authors have suggested an IgE-driven mechanism, based on skin prick test positivity to persulfate salts and the finding of high levels of total serum IgE in hairdressers with OA [4, 6]. However, other data seem to suggest that persulfate salts act through an immunological mechanism without driving an IgE response [7]. Therefore, studies of OA using suitable animal models may be able to shed light on the processes involved in the onset and persistence of bronchial hyperresponsiveness and airway inflammation and remodeling.

In a previous study using local lymph node assays [8], our research group identified ammonium persulfate (AP) as a moderate dermal sensitizer. In later work we developed and validated a mouse model of chemical-induced asthma using AP. In this model, mice were dermally sensitized with AP and then underwent a single airway challenge with AP, which triggered the responses typical of human OA [8, 9].

It has been reported that asthma symptoms and non-specific airway hyperresponsiveness persist even after cessation of exposure. The reason for this is not clear. In the present study, we examined how long the asthmatic response to AP persists after dermal sensitization. The aim of the study was to compare the airway responses, lung inflammation, and immune responses induced by a single intranasal AP challenge administered at variable intervals (between 1 and 90 days) after dermal sensitization to AP.

## Methods

### Mouse model of chemical-induced asthma

On days 1 and 8, male BALB/c mice (~20 g, 6 weeks old; Harlan, The Netherlands) received dermal applications of 5 % ammonium persulfate (AP, [(NH_4_)_2_S_2_O_8_], Sigma-Aldrich, Steinheim, Germany) or vehicle (dimethylsulfoxide (DMSO), Sigma-Aldrich, Steinheim, Germany) on the dorsum of both ears (20 µl). On days 15, 22, 29, 36, 45, 60 and 90, under light anesthesia with isoflurane (Forane^®^, Abbott Laboratories, Madrid, Spain), mice received an intranasal instillation (40 µl) of 1 % AP or vehicle (saline, 0.9 %NaCl). The experimental groups were DMSO/SAL and AP/AP: the first abbreviation identifies the agent used for dermal applications on days 1 and 8 (sensitization) and the second identifies the agent administered via intranasal instillation on days 15, 22, 29, 36, 45, 60 and 90 (challenge). Each group of mice (vehicle or AP) consisted of five to eight animals at each time point. The experiments were repeated twice per group.

### Respiratory responsiveness to methacholine

One day after intranasal challenge, reactivity in response to methacholine was measured using a forced oscillation technique (FlexiVent, SCIREQ, Montreal, Canada). As previously described, mice were anesthetized with pentobarbital (70 mg/kg body weight) (Nembutal ^®^, Abbot Laboratories) and airway resistance (R) was measured using a “snapshot” protocol. For each mouse, R was plotted against methacholine concentration (0–20 mg/ml) and the area under the curve (AUC) was calculated [10].

### Total serum immunoglobulins

After functional airway measurements, mice were deeply anesthetized by an intraperitoneal injection of pentobarbital (90 mg/kg body weight). Blood was taken from the retro-orbital plexus and centrifuged (14,000*g*, 10 min) and serum samples were stored at −80 °C for further analyses. The mouse ELISA kits (Bethyl Laboratories Inc., Montgomery, USA) were used to measure total serum immunoglobulin (Ig)-E, IgG1 and IgG2a (diluted samples 1/5, 1/12,500 and 1/5000 respectively). Measurements were performed according to the manufacturer’s instructions, using biotinylated anti-mouse IgE, IgG1 or IgG2a detection antibodies and horseradish peroxidase conjugate.

### Pulmonary inflammation in bronchoalveolar lavage

Once blood samples were collected, bronchoalveolar lavage (BAL) was performed in situ. The lungs were lavaged three times with 0.7 ml sterile saline (0.9 % NaCl), and the fluid recovered was pooled. Cells were counted using a Bürker hemocytometer (total cells) and the bronchoalveolar lavage (BAL) fluid was centrifuged (1000*g*, 10 min). For differential cell counts, 250 µl of the resuspended cells (100,000 cells/ml) were spun (300*g*, 6 min) (Cytospin 3, Shandon, Thermo Scientific, Cheshire, United Kingdom) onto microscope slides, air-dried and stained [May-Grünwald, 5 min (QCA; Tarragona, Spain) and Giemsa, 15 min (Merck, Darmstadt, Germany)]. For each sample, the numbers of macrophages, eosinophils, neutrophils and lymphocytes were counted in 500 cells.

### Lung pathology

After BAL, lungs were instilled with formaldehyde 3.7–4.0 % until all lobes were deemed to be fully inflated by visual inspection. Instillation was always performed by the same person and in homogeneous conditions. Evaluation of lung injury on slides stained by haematoxylin and eosin (H&E) and Masson’s trichrome was performed by an experienced pathologist in a blinded manner. A semi-quantitative scoring system was used to grade the severity and extent of inflammation, as well as bronchiolar epithelium hyperplasia on H&E stained sections. The thickness of the infiltrate in the interalveolar septa and hyperplasia was graded as follows: 0 (normal) = absence of inflammatory cells; 1 (mild) = 1–2 layers of inflammatory cells; 2 (moderate) = 3–5 layers; 3 (severe) = more than 5 layers [11].

### Data analysis

All data are presented as mean ± standard deviation (SD) and were analysed using the non-parametric Kruskal–Wallis test and Mann–Whitney U-test (Graphpad Prism 4.01, Graphpad Software Inc, San Diego, USA). A level of p < 0.05 (two-tailed) was considered significant.

## Results

### Respiratory responsiveness to methacholine

**Fig. 1:**
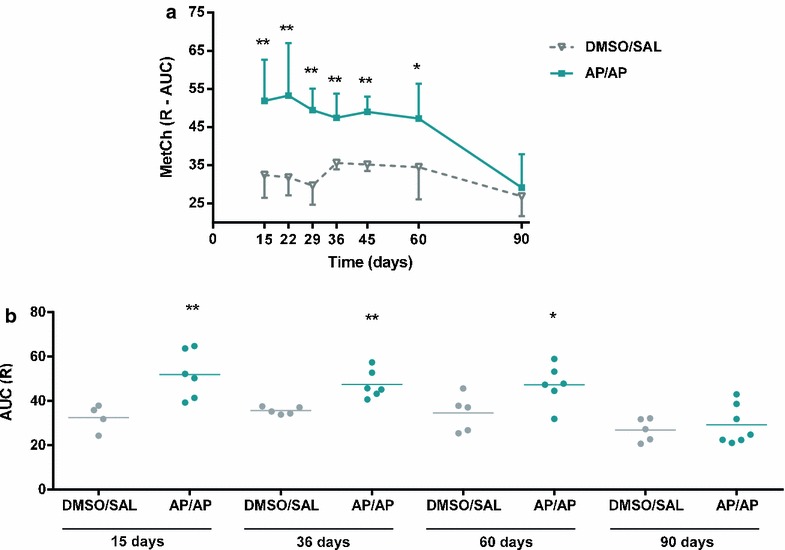
Respiratory responsiveness to methacholine expressed as area under the curve (AUC) of the resistance (R) 24 h after intranasal instillation of AP or vehicle (saline). Experimental groups were DMSO/SAL and AP/AP and were consisted in 5–8 mice per group. First abbreviation refers to dermal sensitization (day 1 and 8), and the second to the agents administered via intranasal instillation (day 15, 22, 29, 36, 45, 60, 90). **a** Mean ± SD of AUC of R against methacholine concentrations (0–20 mg/ml). **b** Mean individual values of AUC at 15, 36, 60 and 90 days after challenge. *p < 0.05, **p < 0.01 compared with DMSO/SAL. *AHR* airway hyperresponsiveness; *AP* ammonium persulfate; *AUC* area under the curve; *DMSO* dimethylsulfoxide; *SAL* saline

The non-specific total respiratory resistance to methacholine increased 24 h after the intranasal instillation with AP in the groups of mice which received dermal treatment with AP and were also intranasally challenged with AP (the AP/AP group) at time points 15, 22, 29, 36, 45 and 60 days (Fig. 1a, b), compared to the control groups which received dermal treatment with DMSO and were intranasally challenged with saline (the DMSO/SAL group), At the last time point, after receiving a single challenge with AP on day 90, no changes in non-specific total respiratory resistance to methacholine were found in the AP/AP mice (Fig. 1a, b).

### Total serum immunoglobulins

**Fig. 2:**
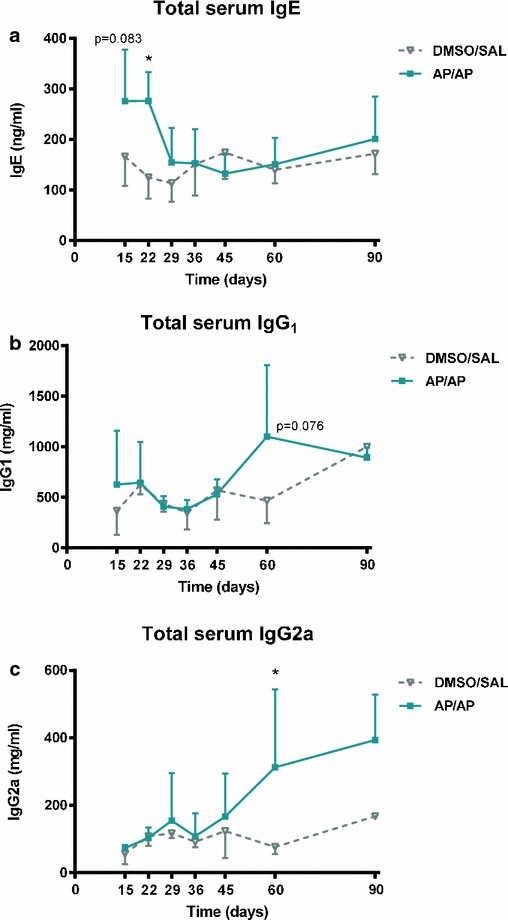
Total serum immunoglobulin (Ig)-E, IgG1 and IgG2a. Blood was collected 24 h after intranasal instillation of AP or vehicle (saline). Total serum IgE, IgG1 and IgG2a were measured using a standard ELISA. Experimental groups are the same as in Fig. 1 and were consisted in 4–6 mice per group. **a** Mean ± SD of total serum IgE. **b** Mean ± SD of total serum IgG1. **c** Mean ± SD of total serum IgG2a. *p < 0.05 compared with DMSO/SAL control group. *AP* ammonium persulfate; *DMSO* dimethylsulfoxide; *SAL* saline

Total serum IgE levels showed a trend towards an increase on day 15 (p = 0.083), and increased significantly on day 22 in the AP/AP group compared with the control mice (Fig. 2a). Total serum IgG1 and IgG2a levels in AP-treated mice started to increase later than total serum IgE. In the case of IgG2a, the increase became significant 60 days after the first dermal sensitization, and was maintained after 90 days; in the case of IgG1 there was a trend towards an increase, although it did not reach significance (p = 0.076) (Fig. 2b, c).

### Pulmonary inflammation (bronchoalveolar lavage)

**Fig. 3:**
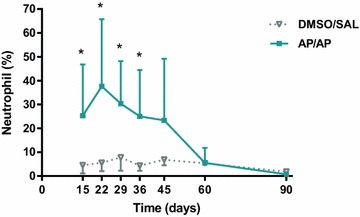
Percentage of neutrophils in BAL obtained 24 h after intranasal instillation of AP or vehicle (saline). Experimental groups are the same as in Fig. 1 and were consisted in 5–8 mice per group. Mean ± SD of percentage of neutrophils in BAL. *p < 0.05 compared with DMSO/SAL control group. No significant differences were found in the other groups studied at different time points. *AP* ammonium persulfate, *BAL* bronchoalveolar lavage, *DMSO* dimethylsulfoxide, *SAL* saline

Figure 3 shows the BAL neutrophil count 1 day after a single challenge. AP-treated mice (AP/AP) showed significantly higher percentages of BAL neutrophils at time points 15, 22, 29, 36 and 45 days than the DMSO/SAL control group (Fig. 3). There were no significant differences in the percentages of eosinophils and lymphocytes in BAL samples between the groups (data not shown).

### Airway histopathology

**Fig. 4:**
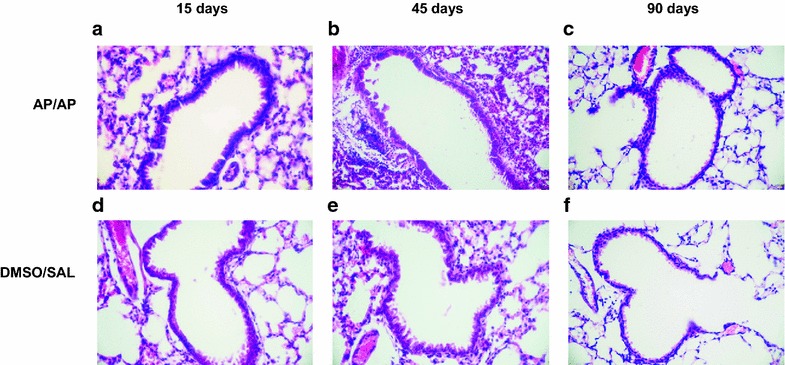
Lung histopathology. Representative images of haematoxylin and eosin stained histological lung sections. Experimental groups in this figure are represented with sections from DMSO/SAL, and AP/AP groups assessed 15 (**a** and **d**), 45 (**b** and **e**) and 90 (**c** and **f**) days after AP sensitization. *AP* ammonium persulfate, *DMSO* dimethylsulfoxide, *SAL* saline

A blinded histopathological examination of lung tissue sections from the AP-treated mice assessed as long as 60 days after sensitization revealed an increase in inflammatory cell infiltration (grade 1–2, mild to moderate) and the presence of alveolar macrophages (grade 1, mild) (Fig. 4a, b) compared with control groups (Fig. 4d, e). At 90 days, the stained sections of AP/AP mice presented reductions in inflammatory cell infiltration (grade 0–1, normal to mild) (Fig. 4c, f). Selectively, at 15 days some moderate peribronchiolar epithelium hyperplasia was observed in the AP/AP group (grade 2, moderate) (Fig. 4a) compared with controls (Fig. 4d). In this acute single challenge model, no collagen deposition was found in the lung sections stained with Masson’s trichrome (data not shown).

## Discussion

We investigated the time course of immunologic and respiratory responses after dermal sensitization in a validated mouse model of OA due to persulfate salts [9]. We were able to induce both respiratory responsiveness to methacholine and pulmonary inflammation in AP-sensitized mice with a single intranasal challenge with AP up to 40 days after initial AP sensitization. Even 60 days after initial AP sensitization, a single challenge could still induce respiratory responsiveness (without neutrophilic inflammation), while 90 days afterwards, a single challenge with AP no longer induced these asthma-like symptoms. In terms of the immune response, there was evidence of systemic sensitization (with an increase in IgE) at early stages, while high IgG levels appeared later.

Exposure to persulfate salts is associated with a high risk of developing OA, although the mechanisms by which these substances induce sensitization and OA are not well understood [2, 4]. It has been suggested that persulfate-induced OA is mediated by an immunological mechanism [2, 4, 12]. Positive skin prick tests to persulfates have been reported, suggesting an IgE-mediated mechanism [4, 12–14]. An increase in total IgE levels has been described in studies with human patients and with mouse models [4, 9]. In this connection, in a mouse model of chemical-induced asthma previously developed by our group using AP [9], we showed that AP, after two dermal applications and only one airway challenge, can induce features of human occupational asthma in mice, including respiratory responsiveness to methacholine, neutrophil inflammation in BAL, T- and B cell proliferation and a Th2 cytokine profile in the auricular lymph nodes (the site of sensitization), and also increased total serum IgE levels.

Recent animal and human data collectively support a central role for skin barrier function and skin exposure in the development of Th2-like sensitization and the subsequent development of asthma. Mouse models have also shown that chemicals can induce mixed Th1/Th2 responses [15, 16]. The skin may play a role both as an important route of exposure and as an immunological organ that can contribute to pulmonary immune diseases [17]. The epidermis contains keratinocytes and Langerhans cells, a major dendritic cell in the skin which can acquire antigen, migrate to draining lymph notes, and initiate immune responses [18]. The activation of dendritic cells with subsequent T-lymphocyte transformation in the lymph nodes draining the skin produces activated effector T-lymphocytes or memory cells in the systemic circulation [19]. Once in the lung, effector T-cells will produce cytokines and chemokines or undertake cytotoxic functions. Repeated lung exposure to the irritant by inhalation doses may synergistically amplify this allergic inflammation and asthma. These cells are able to express high-affinity receptors for IgE and, upon re-exposure, binding of the allergen to IgE orchestrates the immune system to initiate a more aggressive and rapid memory response [20].

In this study, we found a trend towards an increase in total serum IgE levels already at 15 days after initial dermal application of AP. IgE levels remained high until 22 days. A previous study by our group [21] described the course of bronchial hyperresponsiveness and immunologic test results in patients with OA due to persulfate salts, and found that total IgE levels remained increased even in patients who ceased exposure. On the other hand, levels of total serum IgG1 tended to increase from day 60, when total serum IgE levels had returned to baseline values. This is compatible with a Th2 immunological response, despite the unexpected increases in levels of total serum IgG2a (characteristic of a Th1 stimulation in mice) from day 60. These results are consistent with other studies carried out by our group with the same animal model and other mouse models of asthma using LMW agents [9, 11, 20–23], which suggested a mixed Th2-Th1-type immune response in sensitized mice. It has also been proposed that an increase in IgG levels may have a protective effect in this animal model [24], as the percentage of neutrophils decreased at the same time point that IgG started to increase.

Nevertheless, respiratory responsiveness to methacholine still persisted at 45 days despite the increased levels of IgG. Thus, inflammation and respiratory responsiveness were not associated after AP exposure. These results suggest that the presence of abnormal airway smooth muscle function is determinant for respiratory responsiveness to LMW agents in this OA model, while the presence of mucosal airway inflammation may aggravate the situation but is not the cause. Swedin et al. [25] also reported dissociation between airway inflammation and airway hyperresponsiveness (AHR) in an ovalbumin allergic mouse model, suggesting that inflammatory cells in BAL do not change in parallel with AHR. Regarding LMW agents, Vanoirbeek et al. [22] observed the same pattern of dissociation in a mouse model of OA due to isocyanate. These results are consistent with previous observations in subjects with asthma in whom BAL inflammation was not a predictive surrogate marker of AHR [26], suggesting that other factors such as airway wall remodelling, the activation state of inflammatory cells, T-cell activation or autonomic dysfunction may play a more important role in the development of AHR. Recently, in a mouse model of severe asthma, Raundhal et al. [27] demonstrated a role for IFN-ϒ in the induction of AHR, whereas IL-17 promotes neutrophilic airway inflammation. These observations suggest that IFN-ϒ is the predominant cytokine associated with AHR in severe asthma and that airway inflammation and AHR may not always be linked.

The progressive reduction in responsiveness over time also occurs in patients with OA who cease exposure, although the results of the present study only partially reflect this situation. It is well established that patients with OA generally become less responsive to the sensitizer after complete exposure removal [4]. Nevertheless, there is insufficient scientific evidence to assert that cessation of exposure improves asthma symptoms, and many patients do not become completely unresponsive to the sensitizer [28]. In our study, asthma symptoms and functional airway abnormalities had disappeared after 90 days. However, our results did not prove that these mice would not become responsive if repeated exposures to the causal agent were given.

In conclusion, we show that both AHR and airway inflammation responses decrease with increasing time between sensitization and challenge. These findings suggest that dermal contact with a chemical can cause long-term sensitization and may lead to asthmatic symptoms. Moreover, many days after sensitization, exposure to the causal agent may produce various responses of which AHR is the most persistent; for its part, the inflammation response may be decreased. In any case, the mechanisms underlying the process remain undefined and more studies in this direction are needed.
